# Duration-Dependent Caries Risk During Clear Aligner Therapy: A Retrospective Analysis

**DOI:** 10.3390/biomimetics10110786

**Published:** 2025-11-19

**Authors:** Abdurrahman Yalçın, Nursezen Kavasoğlu

**Affiliations:** 1 Department of Restorative Dentistry, Faculty of Dentistry, Batman University, 72000 Batman, Turkey; 2 Department of Orthodontics, Faculty of Dentistry, Batman University, 72000 Batman, Turkey; nursezen.kavasoglu@batman.edu.tr

**Keywords:** clear aligner therapy, dental caries, DMFT index, orthodontics, treatment duration, enamel demineralization

## Abstract

Background: Clear aligner therapy (CAT) represents a biomimetic orthodontic approach that uses flexible thermoplastic materials to reproduce the physiological tooth movement and mechanical load distribution of natural tissues. While these materials promote oral hygiene and aesthetic comfort, their long-term biological impact on the caries process remains uncertain. This retrospective study aimed to evaluate changes in the number of decayed teeth (ΔD) before and after clear aligner treatment and to identify duration-dependent risk factors. Methods: This retrospective study included 362 patients (279 females, 83 males) treated with Invisalign^®^ aligners between 2020 and 2024. Baseline and post-treatment panoramic radiographs were analyzed to determine decayed tooth counts. Age, sex, and total aligner count were recorded. Non-parametric tests, multivariable regression, and ROC analysis were used to assess predictors of ΔD. Results: The mean number of decayed teeth increased slightly from 3.54 ± 2.76 to 3.83 ± 2.93 (*p* < 0.001). Longer treatment duration was independently associated with caries progression (β = +0.0088 per tray, *p* = 0.0037), and each 10-tray increment increased the odds of new decay by 55% (OR = 1.55, 95% CI: 1.26–1.90). ROC analysis identified ≥42 trays as a clinically relevant threshold (AUC = 0.67). Conclusions: Clear aligner therapy demonstrated a statistically significant yet clinically small increase in caries incidence, primarily related to treatment duration. As a biomimetic orthodontic approach that integrates mechanical and biological dynamics, extended clear aligner use may alter biofilm–surface interactions and salivary conditions over time. Therefore, preventive strategies–such as professional fluoride applications, strict cleaning protocols, and shorter recall intervals–should be emphasized for long-duration treatments to preserve the biological benefits of this biomimetic system.

## 1. Introduction

Orthodontic treatment aims to correct dental, skeletal, or combined malocclusions and thereby maintain optimal oral health. Malocclusions are prevalent and encompass a wide range of conditions, including dental crowding, crossbites, and skeletal discrepancies [1,2]. These disorders have traditionally been managed with fixed orthodontic appliances, in which brackets, archwires, and bands are bonded to teeth to achieve controlled movement. Although highly effective, fixed appliances create plaque-retentive niches that complicate oral hygiene practices such as tooth brushing and flossing, thereby elevating the risk of enamel demineralization, white spot lesions (WSLs), and dental caries [3,4,5,6]. Nearly half of orthodontic patients develop enamel opacities or WSLs following treatment, highlighting the high prevalence of appliance-related enamel demineralization [7,8].

In response to increasing aesthetic demands and the desire for improved comfort, clear aligner therapy (CAT) has emerged as a removable and hygienically favorable alternative [9,10]. Compared with traditional fixed appliances, aligners are associated with better oral hygiene, reduced chair time, and lower plaque and gingival indices, and they often cause less discomfort due to their uniform force application [11,12,13]. Beyond clinical parameters, oral microbiome–focused studies show that aligners induce fewer detrimental microbial shifts than fixed appliances, likely because removability facilitates more effective daily biofilm control [14,15,16,17,18]. These findings suggest that CAT may mitigate caries-related risks relative to conventional therapy.

From a biomimetic perspective, clear aligner systems are designed to replicate the natural biomechanics of tooth movement by applying controlled, low-magnitude forces that emulate the adaptive response of periodontal and alveolar tissues [19,20]. The flexible thermoplastic polymers used in these devices distribute mechanical stress in a manner resembling the viscoelastic properties of the periodontal ligament, thereby representing a bioinspired approach to orthodontic force transmission [21]. This biomimetic principle ensures tooth movement that harmonizes with physiological responses while maintaining tissue homeostasis.

Furthermore, the polymeric surfaces of clear aligners exhibit surface energy, wettability, and microbial adhesion properties analogous to enamel-like biomaterials. These bioinspired surface interactions influence bacterial colonization and biofilm formation, directly linking aligner wear to biological processes underlying demineralization and remineralization [22]. Consequently, understanding how prolonged aligner wear modifies these biomimetic interfaces provides valuable insight into the interdisciplinary relationship between material design, biological function, and oral health [23,24].

Nevertheless, clear aligner use is not biologically neutral [11]. Prolonged tray wear (typically 20–22 h per day) and composite attachments can alter the intraoral environment by influencing salivary flow, buffering capacity, and bacterial composition [25,26]. Although multiple reviews have confirmed lower plaque and WSL incidence in aligner users, evidence directly quantifying true caries progression—beyond surrogate measures such as WSLs or gingival indices–remains scarce and sometimes contradictory [27,28]. Some studies suggest that extended aligner use may reduce salivary clearance under the trays, potentially favoring localized acidogenic biofilm formation over time [29]. The duration of orthodontic therapy, particularly the total number of aligners and refinements, thus represents a plausible but underexplored modifier of caries risk.

Given the rapid adoption of CAT and the frequent occurrence of prolonged treatment courses, there is a pressing need for studies that move beyond periodontal surrogates to assess actual caries progression. Therefore, the present retrospective study aimed to quantitatively evaluate changes in the decayed-tooth component of the DMFT index (ΔD) before and after clear aligner therapy and to determine whether aligner count (treatment duration) independently predicts caries progression after adjusting for baseline decay, age, and sex. The null hypothesis was that orthodontic treatment with clear aligners does not significantly alter ΔD following adjustment for these confounders.

## 2. Materials and Methods

### 2.1. Ethical Approval and Study Design

This retrospective observational study was conducted in accordance with the Declaration of Helsinki and approved by the Ethics Committee of Batman University Faculty of Dentistry (Decision No: 2024/08-06; 31 October 2024). The study adhered to the STROBE guidelines for observational studies. Written permission to use anonymized patient data was obtained from the clinical institution prior to data collection. The study design involved pre- and post-treatment comparisons of radiographic caries data in patients who had completed clear aligner therapy. The retrospective design was selected because it allowed evaluation of a large clinical population without additional radiation exposure.

### 2.2. Study Population and Sampling

All consecutive patients treated with Invisalign^®^ clear aligners at Dentalin Oral and Dental Health Polyclinic (Diyarbakır, Turkey) between January 2020 and December 2024 were screened.

Patients were included if: they had completed clear aligner treatment in full, both baseline (pre-treatment) and final (post-treatment) panoramic radiographs were available and diagnostically acceptable, and complete demographic and clinical data (age, sex, aligner count, treatment completion date) were retrievable from electronic records. Exclusion criteria were: absence of initial or final panoramic radiographs, radiographs with motion or exposure artefacts that prevented accurate caries assessment, presence of systemic or periodontal disease that could influence caries activity, ongoing orthodontic treatment at the time of data extraction, use of clear aligner brands other than Invisalign^®^, extraction of permanent teeth for orthodontic reasons, unerupted or partially erupted third molars, and teeth restored solely for aesthetic or Bolton discrepancy corrections.

To ensure adequate representativeness, all eligible cases during the 2020–2024 period were included. No formal power calculation was performed because this was a complete-sample retrospective study rather than a controlled trial.

### 2.3. Data Collection and Variable Definitions

Patient information and radiographs were extracted from the clinic’s electronic database by a single calibrated investigator (N.K.) and verified independently by a second investigator (A.Y.). Data included age (years), sex (male/female), total number of aligners used (treatment duration indicator), and decayed tooth count before (D_0_) and after treatment (D_1_). The primary outcome was the change in decayed teeth (ΔD = D_1_ − D_0_), which represents the difference in the *D* component of the DMFT index between pre- and post-treatment assessments. Secondary variables included baseline D_0_, age, sex, and total number of aligners.

### 2.4. Radiographic Acquisition and Evaluation

All panoramic radiographs were obtained using the same digital unit (Planmeca ProMax^®^, Helsinki, Finland) following a standardized protocol:

- Tube voltage: 66–68 kVp,
- Current: 7–13 mA,
- Exposure time: 16 s,
- Detector resolution: 200 µm,
- Patient positioning: Frankfort horizontal plane parallel to the floor, with bite block and head support to minimize movement.

Images were exported in DICOM format and analyzed in Planmeca Romexis software v3.8.3 (Helsinki, Finland). Radiographs were viewed in a dimly lit environment on a 24-inch diagnostic monitor (1920 × 1080 pixels, brightness 350 cd/m^2^, contrast ratio 1000:1) to ensure optimal visibility of enamel–dentin interfaces. The number of decayed teeth was identified according to WHO criteria for the D component of the DMFT index: radiolucency extending into dentin or the presence of a cavitated surface consistent with active decay. Incipient enamel lesions or white-spot areas without surface breakdown were not counted as decay. All radiographs were evaluated by a single oral and maxillofacial radiologist with seven years of experience who was blinded to treatment duration, sex, and age. To ensure intra-observer reliability, 10% of the images (randomly selected using a computer algorithm) were re-evaluated after two weeks. The Cohen’s κ coefficient was 0.87, indicating excellent agreement. Any discrepancies were resolved by consensus.

Although bitewing radiographs are considered the gold standard for proximal caries detection, panoramic radiographs provide acceptable diagnostic accuracy for the D component of the DMFT index in population-based and retrospective studies. Previous validation studies have shown substantial agreement between panoramic and intraoral radiography [30,31], when using WHO diagnostic thresholds for dentin involvement [32]. This approach minimizes radiation exposure while maintaining reproducibility for large-scale analyses.

### 2.5. Data Management and Quality Control

Data entry and cleaning were performed in IBM SPSS Statistics v25.0 (IBM Corp., Armonk, NY, USA), and all analyses were double-checked for transcription errors. Cases with incomplete or inconsistent information were excluded before statistical processing. Continuous variables were checked for outliers using box-plot visualization and Winsorized if extreme values exceeded 3 interquartile ranges.

### 2.6. Statistical Analysis

Statistical analyses were carried out using IBM SPSS Statistics v25.0 (IBM Corp., Armonk, NY, USA) and R v4.3.2 (R Foundation for Statistical Computing, Vienna, Austria). Shapiro–Wilk and Kolmogorov–Smirnov tests were applied to all continuous variables. Presented as mean ± standard deviation (SD) or median (interquartile range, IQR), and as counts and percentages for categorical variables. The Wilcoxon signed-rank test (non-parametric) was used to compare D_0_ and D_1_ values because ΔD values were not normally distributed. A paired *t*-test was also reported for transparency. Spearman’s rank correlation assessed associations between aligner count and ΔD. ΔD was entered as the dependent variable; aligner count, age, sex, and baseline D_0_ were independent variables. Variance inflation factors (VIFs) confirmed the absence of multicollinearity (VIF < 2). To evaluate factors predicting any increase in caries (ΔD > 0), odds ratios (ORs) and 95% confidence intervals (CIs) were computed. ORs were scaled per 10-tray increment for interpretability. Residuals and leverage plots were examined; heteroskedasticity-consistent (HC3) robust standard errors were used. The receiver operating characteristic curve and Youden index were applied to identify the optimal aligner-count threshold predicting ΔD > 0. The area under the curve (AUC) quantified discriminative ability. A two-tailed *p* < 0.05 was considered statistically significant.

## 3. Results

### 3.1. Baseline Characteristics of the Study Population

A total of 362 patients (279 females [77.1%] and 83 males [22.9%]) who completed clear aligner treatment between 2020 and 2024 met the inclusion criteria and were analyzed. The mean age of the cohort was 29.7 ± 7.9 years, representing a predominantly young adult population. Patients used an average of 36.5 ± 10.2 aligner trays during treatment (range: 14–54), corresponding to typical treatment durations of approximately 7–14 months. Baseline caries prevalence was moderate, with a mean of 3.54 ± 2.76 decayed teeth, which slightly increased to 3.83 ± 2.93 after treatment. The mean change in decayed teeth (ΔD = D_1_ − D_0_) was 0.29 ± 0.64, and the median change was 0 (IQR 0–0). Notably, 72 patients (19.9%) experienced an increase in caries count after therapy, whereas the remaining 80% showed no new decay or a stable status. These values are summarized in Table 1, which details the demographic and baseline clinical characteristics of the study population.

### 3.2. Change in Caries Counts Before and After Clear Aligner Therapy

**Figure 1:**
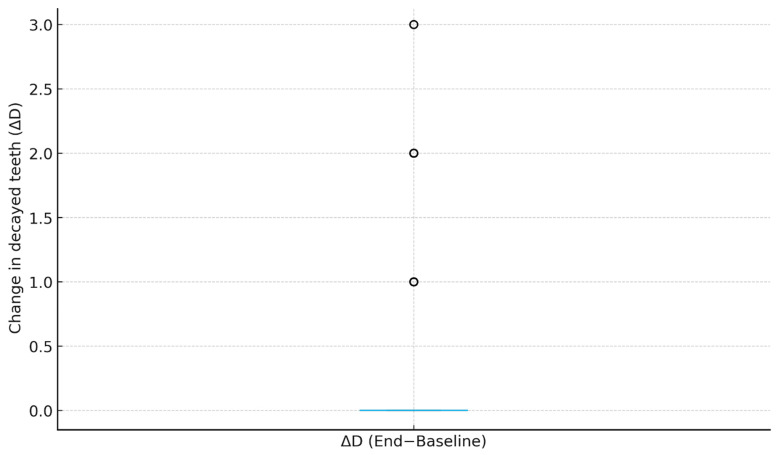
Distribution of ΔD (End–Baseline). Box plot displaying the distribution of change in decayed tooth count (ΔD) across the cohort (*n* = 362). The median is 0 (IQR 0–0), with a small positive shift (mean ΔD = 0.29 ± 0.64), consistent with a statistically significant but clinically small increase after treatment.

Both the Wilcoxon signed-rank test (*p* = 2.63 × 10^−14^) and a confirmatory paired *t*-test (*p* = 2.41 × 10^−16^) revealed a statistically significant increase in the number of decayed teeth following clear aligner therapy. Despite statistical significance, the effect size (Cohen’s d = 0.29) indicated a small clinical magnitude of change. Figure 1 illustrates the distribution of ΔD values, showing that most patients clustered around zero change, while a small subset exhibited a mild positive shift in decay count. This pattern suggests that the overall increase in caries incidence was minor and likely influenced by prolonged treatment duration rather than generalized deterioration of oral hygiene.

### 3.3. Longer Treatment Duration Is Positively Correlated with Caries Progression

**Figure 2:**
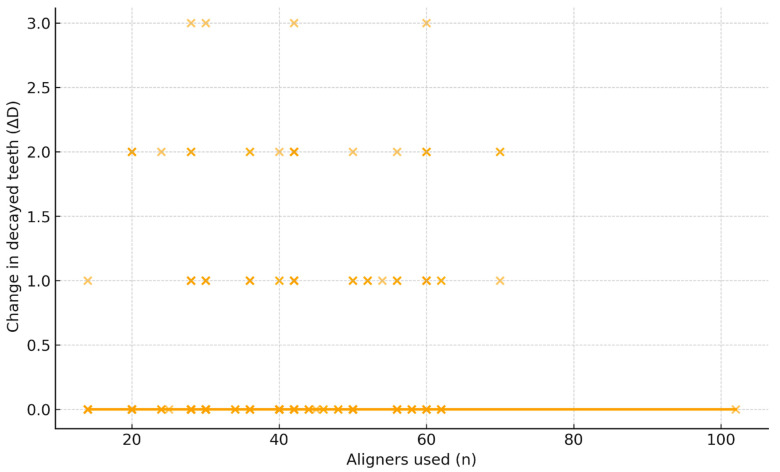
Relationship between ΔD and the number of aligners used. Scatter plot of ΔD versus total aligner count with a LOWESS smoothing curve. A weak–moderate positive association is observed (Spearman ρ = 0.231, *p* = 8.6 × 10^−6^), indicating slightly greater ΔD with longer treatment (more trays). Each dot represents an individual patient’s ΔD value plotted against the total aligner count, and the solid line represents the LOWESS smoothing curve illustrating the overall trend in the data.

Spearman’s rank correlation demonstrated a significant positive relationship between the number of aligners used and the change in decayed teeth (ρ = 0.231, *p* = 8.6 × 10^−6^). As displayed in Figure 2, patients with longer treatment durations tended to exhibit slightly higher ΔD values. Although the correlation strength was modest, it consistently indicated that prolonged therapy–reflected by higher tray counts–was associated with incremental caries progression.

### 3.4. Treatment Duration Remains an Independent Predictor of Caries Increase

To control for potential confounding factors, a multivariable linear regression model was constructed with ΔD as the dependent variable and aligner count, age, sex, and baseline D_0_ as independent variables. After adjustment, aligner count remained a statistically significant predictor of caries progression (β = +0.0088 per tray, *p* = 0.0037). This means that, on average, every additional 10 trays corresponded to approximately +0.088 new decayed teeth, even when age, sex, and baseline decay were held constant. Baseline decay (D_0_) also showed a small but significant positive association (β = +0.027, *p* = 0.025), indicating that patients with higher initial caries levels were slightly more prone to additional decay during treatment. Age and sex were not significant predictors (*p* > 0.05). The adjusted R^2^ = 0.13 suggests that, while treatment duration explains part of the variability in ΔD, other behavioral or biological factors may also contribute. Complete regression outputs are provided in Table 2, along with 95% confidence intervals and robust standard errors.

### 3.5. Each 10-Tray Increment Increases the Odds of New Caries by 55%

To further explore clinical relevance, a binary logistic regression model was applied using ΔD > 0 (any new decay) as the dependent variable. The results confirmed that treatment duration (aligner count) significantly increased the odds of caries progression. Each 10-tray increment raised the likelihood of new decay by 55% (OR = 1.55, 95% CI 1.26–1.90, *p* < 0.001). Baseline decay approached significance (*p* = 0.063), suggesting a possible trend, whereas age and sex remained non-significant (*p* > 0.05). These findings, summarized in Table 3, indicate that treatment duration is a clinically meaningful predictor of caries risk, independent of demographic variables.

### 3.6. ROC Analysis Identifies ≥42 Trays as a Threshold for Increased Caries Risk

**Figure 3:**
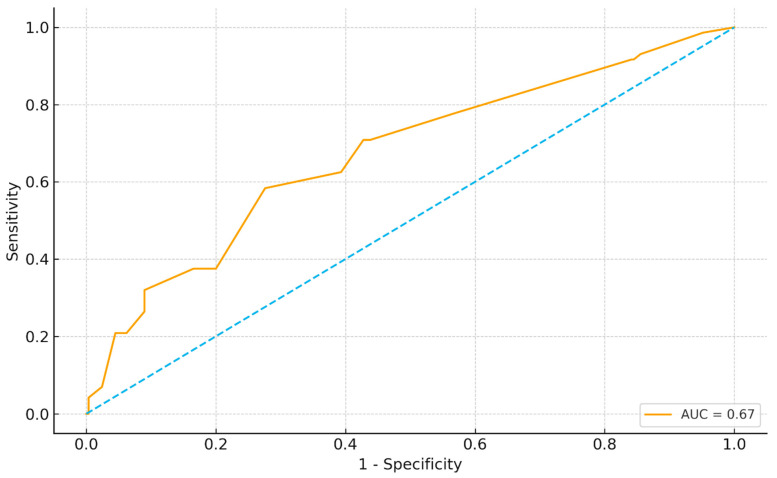
ROC curve for predicting ΔD > 0 using aligner count. Receiver operating characteristic (ROC) curve showing the discriminative ability of total aligner count to predict any increase in decayed teeth (ΔD > 0). Area under the curve (AUC) = 0.67. The Youden-derived optimal threshold is 42 trays (sensitivity = 0.58, specificity = 0.72).

The receiver operating characteristic (ROC) analysis evaluated the discriminative ability of total aligner count to predict any increase in caries (ΔD > 0). The model yielded an area under the curve (AUC) = 0.67, representing fair predictive accuracy. Using the Youden index, an optimal threshold of 42 aligners was identified, corresponding to sensitivity = 0.58 and specificity = 0.72. This means that patients undergoing treatment with ≥42 trays were substantially more likely to experience new caries compared to those below this threshold. Figure 3 presents the ROC curve, highlighting this cut-off point as a clinically relevant risk marker for caries progression during clear aligner therapy.

### 3.7. Representative Radiographs Demonstrate Subtle Post-Treatment Caries Changes

**Figure 4:**
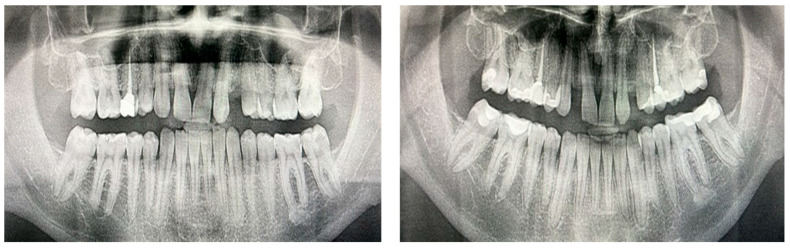
Representative panoramic radiographs of a patient before (**left**) and after clear aligner therapy (**right**). The treatment duration was 12 months (36 aligners). Slight apical resorption was visible on the maxillary incisors post-treatment, a common radiographic finding after orthodontic tooth movement, without clinical relevance.

Representative panoramic radiographs of one patient are displayed in Figure 4, showing the dentition before and after clear aligner therapy. The images exemplify minor increases in decayed areas identifiable on post-treatment films, consistent with the subtle yet statistically significant rise in DMFT (D) scores observed across the sample.

## 4. Discussion

The present study demonstrated a small but statistically significant increase in decayed tooth counts after clear aligner therapy (ΔD = 0.29 ± 0.64). Although the absolute magnitude of change was modest, longer treatment duration, as reflected by total aligner count, was independently associated with greater caries progression after adjustment for age, sex, and baseline decay. Each 10-tray increment raised the odds of developing new caries (ΔD > 0) by 55%, and receiver-operating-characteristic (ROC) analysis identified 42 trays as a practical threshold predicting caries increase (AUC = 0.67). However, this AUC indicates only fair, not strong, discriminative power; therefore, the 42-tray threshold should be viewed as an approximate clinical indicator rather than a validated cutoff. Although statistically significant, the mean ΔD of 0.29 teeth represents a clinically small effect–roughly one additional decayed surface per three to four long-term patients. Hence, treatment duration predicts measurable but limited risk, which should be interpreted within the context of individual caries susceptibility and preventive-care adherence.

Previous systematic reviews and randomized trials consistently show that clear aligner therapy promotes better oral hygiene, lower plaque accumulation, and reduced incidence of white-spot lesions (WSLs) compared with fixed appliances [27,33,34,35,36,37]. These studies largely examined short-term outcomes or surrogate parameters of demineralization rather than true caries progression. Our findings diverge by providing radiographic evidence of incremental decay with prolonged aligner use, despite the overall hygiene advantage. This nuance refines the existing consensus: while aligners are less plaque-retentive, extended wear may gradually diminish their protective effect.

From a biological standpoint, the results are plausible. Prolonged tray wear creates a semi-closed intraoral microenvironment that restricts salivary exchange and buffering capacity, favoring localized acid production by cariogenic microorganisms. Over months, this reduced fluid dynamics can promote the persistence of acidogenic biofilms dominated by *Streptococcus mutans* and *Lactobacillus* species [22,38,39]. Such microecological changes may explain the observed positive correlation between treatment duration and ΔD, highlighting how the very biomimetic property of tight surface adaptation–advantageous for controlled tooth movement–can become a biological trade-off when exposure is prolonged.

Clinically, identifying a ≥42-tray threshold offers a pragmatic cue for preventive intervention. Patients with longer treatment plans and higher baseline caries experience should receive intensified hygiene reinforcement, topical fluoride varnish or high-fluoride toothpaste regimens, and shorter recall intervals during mid-to-late treatment phases [40,41]. The concept of “risk-based recall” is particularly valuable in adult orthodontic patients who often present with restorations or enamel defects that further predispose them to demineralization [22,42]. Translating statistical thresholds into individualized recall strategies bridges the gap between quantitative modeling and clinical decision-making.

This study’s strengths include a relatively large sample size, pre-post design, standardized radiographic assessment, and multivariable modeling adjusted for baseline decay, age, and sex. The integration of ROC-derived clinical markers enhances translational relevance by linking statistical performance with actionable risk stratification.

Limitations include its retrospective single-center design and reliance on panoramic radiographs, which, although standardized and practical, offer lower sensitivity than bitewing imaging for early enamel lesions. Nevertheless, panoramic radiography yields acceptable diagnostic accuracy for the D-component of the DMFT index in population-based studies and has been validated in WHO-based protocols [30,31,32]. Additionally, unmeasured confounders such as diet, oral-hygiene behavior, fluoride exposure, and aligner-wear compliance may have influenced caries development. Their absence could partly explain the residual variability in ΔD and modestly overestimate the effect of treatment duration. Despite these constraints, consistent imaging parameters, blinded evaluation, and robust statistical corrections strengthen internal validity.

Future research should employ prospective, multicenter designs to confirm the duration-dependent relationship between aligner wear and caries progression. Incorporating standardized diagnostic systems (e.g., ICDAS or QLF), salivary and microbiological biomarkers, and objective wear-time tracking devices will clarify the biological mechanisms underlying prolonged aligner use. Moreover, integrating behavioral and compliance data will permit more accurate modeling of true caries risk, thereby refining preventive protocols within this biomimetic orthodontic framework. Such evidence-based refinements are essential to optimize long-term outcomes and preserve the biological advantages of clear aligner therapy.

## 5. Conclusions

Clear aligner therapy was associated with a small but statistically significant increase in caries incidence, mainly related to treatment duration. However, the biomimetic design of aligners such as mimicking physiological tooth movement and biocompatible surface interaction remains a valuable advancement in orthodontics. Long-term clinical success depends on balancing these biological advantages with personalized preventive care. Future prospective and multicenter studies should clarify the biological mechanisms linking prolonged wear duration and caries risk, ultimately optimizing this biomimetic approach for sustainable oral health.

## Figures and Tables

**Table 1:** Baseline characteristics of patients treated with clear aligners.

Characteristic	Value
Sample size	362
Female, *n* (%)	279 (77.1%)
Male, *n* (%)	83 (22.9%)
Age (mean ± SD, years)	29.7 ± 7.9
Baseline decayed (mean ± SD)	3.54 ± 2.76
End decayed (mean ± SD)	3.83 ± 2.93
Δ Decayed (End–Baseline, mean ± SD)	0.29 ± 0.64
Aligners used (mean ± SD)	36.5 ± 10.2
Increase > 0 *n* (%)	72 (19.9%)

Descriptive statistics of the study population treated with Invisalign^®^ clear aligners between 2020 and 2024. Values are presented as mean ± standard deviation (SD) or number (percentage).

**Table 2:** Multivariable linear regression for ΔD (End–Baseline decayed teeth).

Predictor	β (Per Unit)	95% CI	*p*-Value
Intercept	-	-	-
Aligners (per 1 tray)	+0.0088	0.003 to 0.015	0.0037
Age (per 1 year)	+0.0015	−0.001 to 0.004	0.214
Male (vs. Female)	+0.0370	−0.001 to 0.076	0.053
Baseline decayed (D_0_, per 1 tooth)	+0.0270	0.004 to 0.050	0.025

Model diagnostics: Adjusted R^2^ = 0.13 (robust SE, HC3). Multiple linear regression model with ΔD as the dependent variable and aligner count, age, sex, and baseline decay (D_0_) as predictors. Positive β values indicate greater increases in decayed teeth with higher predictor levels. Robust (HC3) standard errors were used; statistical significance set at *p* < 0.05.

**Table 3:** Binary logistic regression predicting caries progression (ΔD > 0).

Predictor	OR	95% CI	*p*-Value
Aligners (per 10 trays)	1.55	1.26–1.90	<0.001
Age (per 1 year)	1.02	0.97–1.08	0.312
Male (vs. Female)	1.32	0.94–1.84	0.106
Baseline decayed (D_0_, per 1 tooth)	1.19	0.98–1.44	0.063

Logistic regression with outcome ΔD > 0 (any increase in decayed tooth count from baseline to end of treatment). Odds ratios (ORs) and 95% confidence intervals (CIs) are adjusted for all listed predictors. OR for aligners is scaled per 10-tray increment for clinical interpretability. Statistical significance set at *p* < 0.05.

## Data Availability

All generated data is supplied within current study. Further inquiries should be addressed to the corresponding author.

## References

[1] Jamilian A., Kiaee B., Sanayei S., Khosravi S., Perillo L. (2016). Orthodontic Treatment of Malocclusion and its Impact on Oral Health-Related Quality of Life. Open Dent. J..

[2] Shen Y., Jiang X., Yu J. (2023). The combined orthodontic and restorative treatment for patients with malocclusion and dentition defects: A randomized controlled trial. Medicine.

[3] Karkhanechi M., Chow D., Sipkin J., Sherman D., Boylan R.J., Norman R.G., Craig R.G., Cisneros G.J. (2013). Periodontal status of adult patients treated with fixed buccal appliances and removable aligners over one year of active orthodontic therapy. Angle Orthod..

[4] Tamer İ., Öztaş E., Marşan G. (2019). Orthodontic Treatment with Clear Aligners and The Scientific Reality Behind Their Marketing: A Literature Review. Turk. J. Orthod..

[5] Sabatini S., Castaldi M., De Stefano A.A., Galluccio G., Grassi R., Nardi G.M. (2025). Protocols and Technologies Used by Italian Dental Professionals to Maintain Good Oral Health in Orthodontic Patients Before, During and After Treatment: A Survey Study. Oral.

[6] Julien K.C., Buschang P.H., Campbell P.M. (2013). Prevalence of white spot lesion formation during orthodontic treatment. Angle Orthod..

[7] Toti Ç., Meto A., Kaçani G., Droboniku E., Hysi D., Tepedino M., Zaja E., Fiorillo L., Meto A., Buci D. (2022). White Spots Prevalence and Tooth Brush Habits during Orthodontic Treatment. Healthcare.

[8] Guzmán-Armstrong S., Chalmers J., Warren J.J. (2010). White spot lesions: Prevention and treatment. Am. J. Orthod. Dentofac. Orthop..

[9] Sardana D., Schwendicke F., Kosan E., Tüfekçi E. (2023). White spot lesions in orthodontics: Consensus statements for prevention and management. Angle Orthod..

[10] Wu Y., Cao L., Cong J. (2020). The periodontal status of removable appliances vs fixed appliances: A comparative meta-analysis. Medicine.

[11] AlMogbel A. (2023). Clear Aligner Therapy: Up to date review article. J. Orthod. Sci..

[12] Pereira D., Machado V., Botelho J., Proença L., Mendes J.J., Delgado A.S. (2020). Comparison of Pain Perception between Clear Aligners and Fixed Appliances: A Systematic Review and Meta-Analysis. Appl. Sci..

[13] Albhaisi Z., Al-Khateeb S.N., Abu Alhaija E.S. (2020). Enamel demineralization during clear aligner orthodontic treatment compared with fixed appliance therapy, evaluated with quantitative light-induced fluorescence: A randomized clinical trial. Am. J. Orthod. Dentofac. Orthop..

[14] Dipalma G., Marinelli G., Inchingolo F., Longo M., Di Giulio Cesare M., Di Serio S., Palermo A., Del Fabbro M., Inchingolo A.D., Inchingolo A.M. (2025). Clinical Efficacy of Clear Aligners in Class II Malocclusion: From Pediatric to Adult Cases-A Narrative Review. J. Funct. Biomater..

[15] Dallel I., Ben Salem I., Merghni A., Bellalah W., Neffati F., Tobji S., Mastouri M., Ben Amor A. (2020). Influence of orthodontic appliance type on salivary parameters during treatment. Angle Orthod..

[16] España-Pamplona P., Bernés-Martínez L., Andrés-Castelló C., Bolás-Colveé B., Adobes-Martín M., Garcovich D. (2024). Changes in the Oral Microbiota with the Use of Aligners vs. Braces: A Systematic Review. J. Clin. Med..

[17] Giannini L., Galbiati G., Tartaglia F.C., Grecolini M.E., Maspero C., Biagi R. (2025). Orthodontic Treatment with Fixed Appliances Versus Aligners: An Experimental Study of Periodontal Aspects. Dent. J..

[18] Meto A., Colombari B., Castagnoli A., Sarti M., Denti L., Blasi E. (2019). Efficacy of a Copper-Calcium-Hydroxide Solution in Reducing Microbial Plaque on Orthodontic Clear Aligners: A Case Report. Eur. J. Dent..

[19] Bichu Y.M., Alwafi A., Liu X., Andrews J., Ludwig B., Bichu A.Y., Zou B. (2023). Advances in orthodontic clear aligner materials. Bioact. Mater..

[20] Elshazly T.M., Bourauel C., Ismail A., Ghoraba O., Aldesoki M., Salvatori D., Elattar H., Alhotan A., Alkabani Y. (2024). Effect of material composition and thickness of orthodontic aligners on the transmission and distribution of forces: An in vitro study. Clin. Oral Investig..

[21] Cenzato N., Di Iasio G., Martìn Carreras-Presas C., Caprioglio A., Del Fabbro M. (2024). Materials for Clear Aligners—A Comprehensive Exploration of Characteristics and Innovations: A Scoping Review. Appl. Sci..

[22] Martínez Gil-Ortega A., Paz-Cortés M.M., Viñas M.J., Cintora-López P., Martín-Vacas A., Gil J., Aragoneses J.M. (2025). Effect of Surface Wettability and Energy on Bacterial Adhesion to Dental Aligners: A Comparative In Vitro Study. Bioengineering.

[23] Tektas S., Thurnheer T., Eliades T., Attin T., Karygianni L. (2020). Initial Bacterial Adhesion and Biofilm Formation on Aligner Materials. Antibiotics.

[24] Suter F., Zinelis S., Patcas R., Schätzle M., Eliades G., Eliades T. (2020). Roughness and wettability of aligner materials. J. Orthod..

[25] Chen W., Chen J., Bai D., Wang P., Shu R. (2024). Effects of clear aligners and traditional removable appliances on oral microbiome in mixed dentition: A comparative study. BMC Oral Health.

[26] Ponton E., Rossouw P.E., Javed F. (2025). Comparison of Oral Microbial Profile Among Patients Undergoing Clear Aligner and Fixed Orthodontic Therapies for the Treatment of Malocclusions: An Updated Review. Dent. J..

[27] Raghavan S., Abu Alhaija E.S., Duggal M.S., Narasimhan S., Al-Maweri S.A. (2023). White spot lesions, plaque accumulation and salivary caries-associated bacteria in clear aligners compared to fixed orthodontic treatment. A systematic review and meta- analysis. BMC Oral Health.

[28] Di Spirito F., D’Ambrosio F., Cannatà D., D’Antò V., Giordano F., Martina S. (2023). Impact of Clear Aligners versus Fixed Appliances on Periodontal Status of Patients Undergoing Orthodontic Treatment: A Systematic Review of Systematic Reviews. Healthcare.

[29] Çetin S., Akdeniz B.S. (2025). Comparative Study of Proximal Caries Formation and Decay, Missing, Filled Teeth Scores in Clear Aligners and Fixed Orthodontic Treatments. Turk. J. Orthod..

[30] Wenzel A. (2021). Radiographic modalities for diagnosis of caries in a historical perspective: From film to machine-intelligence supported systems. Dentomaxillofac. Radiol..

[31] Keenan J.R., Keenan A.V. (2016). Accuracy of dental radiographs for caries detection. Evid.-Based Dent..

[32] World Health Organization (2013). Oral Health Surveys: Basic Methods.

[33] Buschang P.H., Chastain D., Keylor C.L., Crosby D., Julien K.C. (2019). Incidence of white spot lesions among patients treated with clear aligners and traditional braces. Angle Orthod..

[34] Niu Q., Chen S., Bai R., Lu Y., Peng L., Han B., Yu T. (2024). Dynamics of the oral microbiome during orthodontic treatment and antimicrobial advances for orthodontic appliances. iScience.

[35] Jiang Q., Li J., Mei L., Du J., Levrini L., Abbate G.M., Li H. (2018). Periodontal health during orthodontic treatment with clear aligners and fixed appliances: A meta-analysis. J. Am. Dent. Assoc..

[36] Mishra S., Swarnakar S., Nanda S.B. (2024). Evaluation of white spot lesion (WSL) and periodontal (PDL) health between patients treated with clear aligners and conventional fixed orthodontic brackets: A systematic review. J. Contemp. Orthod..

[37] Oikonomou E., Foros P., Tagkli A., Rahiotis C., Eliades T., Koletsi D. (2021). Impact of Aligners and Fixed Appliances on Oral Health during Orthodontic Treatment: A Systematic Review and Meta-Analysis. Oral Health Prev. Dent..

[38] Rouzi M., Zhang X., Jiang Q., Long H., Lai W., Li X. (2023). Impact of Clear Aligners on Oral Health and Oral Microbiome During Orthodontic Treatment. Int. Dent. J..

[39] Marya A., Porntaveetus T., Okazaki K., Jamilian A. (2025). A comparative review of the oral microbiome in clear aligners and fixed orthodontic appliances. Evid.-Based Dent..

[40] Baik A., Alamoudi N., El-Housseiny A., Altuwirqi A. (2021). Fluoride Varnishes for Preventing Occlusal Dental Caries: A Review. Dent. J..

[41] Zhou Y., Wang Y., Wang X., Volière G., Hu R. (2014). The impact of orthodontic treatment on the quality of life a systematic review. BMC Oral Health.

[42] Garcia-Torres F., Jurado C.A., Rojas-Rueda S., Sanchez-Vazquez S., Floriani F., Fischer N.G., Tsujimoto A. (2024). Combining Orthodontic and Restorative Care with Novel Workflows. Dent. J..

